# Improved Vaccine against PRRSV: Current Progress and Future Perspective

**DOI:** 10.3389/fmicb.2017.01635

**Published:** 2017-08-28

**Authors:** Yuchen Nan, Chunyan Wu, Guoqian Gu, Weiyao Sun, Yan-Jin Zhang, En-Min Zhou

**Affiliations:** ^1^Department of Preventive Veterinary Medicine, College of Veterinary Medicine, Northwest A&F University Yangling, China; ^2^Molecular Virology Laboratory, Virginia-Maryland College of Veterinary Medicine and Maryland Pathogen Research Institute, University of Maryland, College Park MD, United States

**Keywords:** porcine reproductive and respiratory syndrome virus, PRRSV, PRRSV vaccine, modified live virus, interferon, cross-protection

## Abstract

Porcine reproductive and respiratory syndrome virus (PRRSV), one of the most economically significant pathogens worldwide, has caused numerous outbreaks during the past 30 years. PRRSV infection causes reproductive failure in sows and respiratory disease in growing and finishing pigs, leading to huge economic losses for the swine industry. This impact has become even more significant with the recent emergence of highly pathogenic PRRSV strains from China, further exacerbating global food security. Since new PRRSV variants are constantly emerging from outbreaks, current strategies for controlling PRRSV have been largely inadequate, even though our understanding of PRRSV virology, evolution and host immune response has been rapidly expanding. Meanwhile, practical experience has revealed numerous safety and efficacy concerns for currently licensed vaccines, such as shedding of modified live virus (MLV), reversion to virulence, recombination between field strains and MLV and failure to elicit protective immunity against heterogeneous virus. Therefore, an effective vaccine against PRRSV infection is urgently needed. Here, we systematically review recent advances in PRRSV vaccine development. Antigenic variations resulting from PRRSV evolution, identification of neutralizing epitopes for heterogeneous isolates, broad neutralizing antibodies against PRRSV, chimeric virus generated by reverse genetics, and novel PRRSV strains with interferon-inducing phenotype will be discussed in detail. Moreover, techniques that could potentially transform current MLV vaccines into a superior vaccine will receive special emphasis, as will new insights for future PRRSV vaccine development. Ultimately, improved PRRSV vaccines may overcome the disadvantages of current vaccines and minimize the PRRS impact to the swine industry.

## Introduction

Porcine reproductive and respiratory syndrome virus (PRRSV) is a positive-stranded enveloped RNA virus which belongs to the genus *Arterivirus*, family *Arteriviridae* and order *Nidovirales* (Lunney et al., 2016). The genome size of PRRSV is about 15 kb and is organized with replicase genes located at the 5′ end followed by the genes encoding structural proteins toward the 3′ end (Snijder and Meulenberg, 1998; Dokland, 2010). The genome of PRRSV contains over 10 open reading frames (ORFs). ORF1a and ORF1b account for over two thirds of the viral genome and encode non-structural proteins that are necessary for viral replication (Lunney et al., 2016), while ORFs 2-7 encode structural proteins (Lunney et al., 2016). There are two well recognized PRRSV genotypes: Type 1, or European-like (prototype Lelystad) and Type 2, or North American-like (prototype VR-2332) (Wensvoort et al., 1991; Mardassi et al., 1994). Recently, PRRSV Type 1 and Type 2 were classified into two species in the genus *Porartevirus*: *PRRSV-1* and *PRRSV-2*, respectively, in the new taxonomy (Adams et al., 2016; Kuhn et al., 2016).

*PRRSV-1* and *PRRSV-2* strains share approximately 60% nucleotide sequence identity and exhibit serotype differences (van Woensel et al., 1998; Forsberg, 2005). However, overall disease phenotype, gross clinical signs, genomic organization and temporal emergence are all similar between the two species (Kappes and Faaberg, 2015). Unlike other members of *Arterivirus*, which have relatively broad tropisms for cells of various origins (Zhang and Yoo, 2015), PRRSV infection is highly restricted to cells of the monocyte-macrophage lineage, such as porcine alveolar macrophages (PAMs) (Albina et al., 1998; Morgan et al., 2014), macrophages from the spleen, tonsils, lymph nodes, liver, Peyer’s patches and thymus, as well as peritoneal macrophages from blood and progenitor cells in bone marrow (Sur et al., 1996; Duan et al., 1997a,b; Wang et al., 2016). Moreover, bone marrow-derived dendritic cells and macrophages are susceptible to PRRSV infection *in vitro* as well (Chang et al., 2008; Chaudhuri et al., 2016). Generally, only PAMs in lung are considered to be the primary target of PRRSV *in vivo* (Albina et al., 1998; Morgan et al., 2014).

Numerous studies have demonstrated that PRRSV infection is mediated by various cellular receptors or factors (Shi et al., 2015) such as heparin sulfate (HS) (Delputte et al., 2002), vimentin (Kim et al., 2006), CD151 (Wu et al., 2014), porcine CD163 (CD163) (Guo et al., 2014), sialoadhesin (CD169) (Delputte et al., 2007), DC-SIGN (CD209) (Huang et al., 2009; Pineyro et al., 2016), and MYH9 (Gao et al., 2016). A list of receptors utilized by PRRSV was summarized as **Table 1**. However, only CD163 is indispensable for PRRSV infection both *in vitro* and *in vivo* (Burkard et al., 2017). In addition to PAMs, immortalized cell lines commonly used for *in vitro* PRRSV propagation are sub-clones derived from the African green monkey kidney cell line MA104, such as MARC-145, CRL11171 and CRL2621a. While MARC-145 cells are predominantly used in academic laboratories (Benfield et al., 1992; Meng et al., 1996). Moreover, several cell lines from various species after introduction of CD163 cDNA, such as PK-15, CRL2843, HEK293T and BHK21, have been shown to support PRRSV replication as well (Calvert et al., 2007; Delrue et al., 2010; Wang et al., 2013d).

**Table 1:** List cellular receptors of PRRSV and their functions during PRRSV infection.

Receptor name	Function during virus infection	Interacting counterpart from PRRSV virion
Heparin sulfate	Initial PRRSV attachment	Disulfide-linked M/GP5 complex
Vimentin	Opsonize and endocytosis of PRRSV virion	Nucleocapsid protein
CD151	Unknown	3’ untranslated region (UTR) RNA of PRRSV
CD163	PRRSV entry	GP2a and GP4
CD169 (Sialoadhesin)	Virion attachment and endocytosis	Sialic acids on the surface of PRRSV GP5 protein
CD209 (DC-SIGN)	Unknown	Unknown
MYH9	Endocytosis of PRRSV virion	GP5

Current control of PRRS is inadequate despite substantial efforts have been dedicated to minimize the impact of this disease. Since the first report of PRRSV in the United States in 1987, PRRSV remains one of the major challenges for the swine industry globally and continuously evolves to cause new outbreaks (Tian et al., 2007; Rowland et al., 2012). Although the first vaccine, Ingelvac PRRS^®^ MLV, has been commercially available and widely used for more than two decades, the prevalence of PRRSV infection in swine herds is still high and vaccination has demonstrated only limited control of PRRS (Butler et al., 2014). Indeed, this dilemma of PRRSV vaccine development has been somewhat surprising, since a number of vaccines against equine arteritis virus (EAV, another member of the genus *Arterivirus*) are available and are highly effective (Balasuriya and MacLachlan, 2004).

Porcine reproductive and respiratory syndrome virus can replicate in numerous cell types from a variety of species as long as the cells express functional porcine CD163 (Van Breedam et al., 2010). However, swine (except wild boar) is the only natural host for PRRSV and there is no evidence demonstrating cross-species infection. This situation is reminiscent of smallpox infection in humans or rinderpest infection in cattle, which have been successfully controlled through vaccination. Therefore, in an analogous manner, it is hoped that soon large-scale immunization with highly effective vaccines will finally eliminate PRRSV from swine herds. In fact, a highly effective vaccine of another swine RNA virus, such as C strain MLV for classic swine fever virus (CSFV), is characterized by its genetic stability and safety to pigs of all ages, as well as its ability to induce sterile immunity and provide rapid, long-lasting and complete protection against CSFV of various genotypes (Luo et al., 2014).

In 2007, the Colloquium on Prospects for Development of an Effective PRRSV Virus Vaccine was held at the University of Illinois, College of Veterinary Medicine, United States to discuss the state of current knowledge about PRRS vaccination (Rock, 2007). All attendees, including experts in PRRS, virology, immunology and vaccinology, as well as clinical veterinarians, academics and vaccine industry scientists set new standards for the next generation of PRRSV vaccines. These standards include rapid induction of immunity, protection against most currently prevalent PRRSV strains, no adverse outcomes to swine health and ability to differentiate vaccinated from infected animals (Rock, 2007). However, although almost 10 years have passed since that meeting, no new vaccine candidates are commercially available that meet all of the above criteria.

In recent years, multiple techniques have been used to develop new PRRSV vaccines and some novel discoveries have been made with the potential to transform current PRRSV vaccinology. In this review, we summarize both the progress and challenges faced by current PRRSV vaccine researchers and provide new insights to guide future efforts.

## PRRSV Constantly Evolves to Cause New Outbreaks and Becomes More Virulent

Although the origin of PRRSV is still a myth, initial outbreak of PRRS was reported nearly simultaneous both in North America (1987) and Western Europe (1990) (Goyal, 1993). Since then, this disease has rapidly emerged across the rest of the world (Lunney et al., 2016). However, since the identification and characterization of prototype PRRSVs for both *PRRSV-1* and *PRRSV-2*, new variants of PRRSVs have constantly evolved and appeared in outbreaks with increasingly divergent and virulent phenotypes (Kappes and Faaberg, 2015).

Notably, retrospective studies have suggested that PRRSV infection existed for years in swine herds globally before the official recognition of PRRS by practitioners of swine industry. In a serologic study conducted in Ontario, Canada, screening was performed to detect PRRSV antibody-positive samples from pig sera collected between 1978 and 1982, prior to the 1987 outbreak. Among those samples, the earliest positive sample for PRRSV antibodies was identified as early as 1979, with increasing PRRSV-positive frequencies detected in later samples (Carman et al., 1995). In Iowa, United States, similar studies detected PRRSV infection in swine herds during or shortly prior to 1985 (Zimmerman et al., 1997). Moreover, similar retrospective screenings in Europe and Asia have also confirmed the presence of PRRSV in local swine herds prior to the first known outbreak and PRRSV antibody-positive ratio in tested samples increased rapidly in a very short time frame before the emergence of major clinical disease outbreaks (Zimmerman, 2003). In other words, these retrospective investigations suggested that non-pathogenic ancestral strains for both PRRSV genotypes had been circulating in swine herds in the pre-PRRS era for years before becoming pathogenic and appearing in outbreaks since 1987.

In addition to non-pathogenic PRRSV strains circulating before the first outbreak, VR2385, a virulent *PRRSV-2* strain identified in the mid-1990s, was isolated from PRRSV-infected herds soon after the identification of the *PRRSV-2* prototype strain (ATCC VR2332) and diverged from VR2332 about 8% in nucleotide identity (Meng et al., 1996). Lately in 1998, another atypical PRRSV strain emerged and caused high fetal mortality and abortion in vaccinated herds in the United States (Mengeling et al., 1998). Subsequently, since 2001 many virulent isolates belonging to the same group of viruses (characterized by restriction fragment length polymorphism type 1-8-4) have been identified, leading to the discovery of the highly virulent MN184 strain, which is quite distinct (>14.5% nucleotide difference) from other genotype 2 strains (Han et al., 2006). In 2006, the key event reforming the concept of PRRSV pathogenesis was the emergence of a highly pathogenic PRRSV strain (now recognized as HP-PRRSV) with a unique molecular marker (deletion of 30 amino acids in nsp2) and high mortality rate (20–100%) in sows in South China and North Vietnam (Tian et al., 2007) and later to Southeast Asia and India (An et al., 2011; Rajkhowa et al., 2015). Additionally, since the identification of genotype 2 strain NADC30 in the United States in 2008 (Brockmeier et al., 2012), NADC30-like strains (with mortality rates of 30–50%) were soon isolated across China as well (Zhou et al., 2015; Li et al., 2016). Challenge experiments with NADC30-like virus suggested that almost all commercial vaccines licensed in China confer limited protection (Bai et al., 2016).

Although less attention has been paid to *PRRSV-1* as it was previously thought to be less divergent than *PRRSV-2*, accumulating evidence suggests that its genetic diversity is actually similar to that of *PRRSV-2*. Moreover, *PRRSV-1* has evolved in the same direction as *PRRSV-2* to become more pathogenic (Forsberg et al., 2002; Morgan et al., 2013). In 2010, the hallmark of *PRRSV-1* evolution was identification of the Lena strain (isolated in 2007), a highly pathogenic strain which shares 87% nucleotide sequence identity with the *PRRSV-1* prototype Lelystad virus. This is a similar degree of identity as observed between *PRRSV-2* strains MN184 and VR2332 (Karniychuk et al., 2010). In spite of its similarity to the *PRRSV-1* prototype virus, Lena infection cannot be fully prevented by attenuated European subtype 1 vaccine (Porcilis^®^ PRRS, Merck) (Trus et al., 2014). Notably, spontaneous deletion of nsp2 has also been observed in PRRSV-1 Lena strains as well (Karniychuk et al., 2010; Trus et al., 2014).

The high degree of observed variability suggests that both PRRSV-1 and PRRSV-2 are constantly evolving to adapt to existing immunity and re-emerging as new variants to cause new outbreaks continuously (Morgan et al., 2013). Although the molecular mechanism of PRRSV evolution is still not fully understood, both epidemiological and molecular evolution data point to a time of divergence between PRRSV-1 and PRRSV-2 of approximately 1980. If this estimate is correct, PRRSV has evolved at the highest evolutionary rate (on the order of 10^-2^/site/year) of all known RNA viruses (with rates ranging from 10^-3^ to 10^-5^/site/year) (Hanada et al., 2005). Taking all information together, including outbreaks of PRRSV in vaccinated herds, epidemiological monitoring data and molecular evolutionary analysis, it appears that PRRSV is constantly evolving to cause new outbreaks and is becoming more virulent with ability to evade vaccine-induced immunity. Therefore, creating an effective vaccine to target constantly evolving PRRSV is a top priority for controlling PRRS.

## Current Licensed PRRSV Vaccines and Vaccines Under Development

### Modified Live Virus (MLV) Vaccines against PRRSV

Since the discovery of PRRSV, several MLV vaccines have been launched against both PRRSV-1 and PRRSV-2 and licensed in various countries depending on circulating viral genotypes. Porcilis^®^ PRRS (Merck), Ingelvac PRRSFLEX^®^ EU (Boehringer Ingelheim), Amervac-PRRS (Hipra), Pyrsvac-183 (Syva) and ReproCyc^®^ PRRS EU (Boehringer Ingelheim) were developed against PRRSV-1 and are mainly licensed in West European countries and other countries with PRRSV-1 prevalence. Ingelvac PRRS^®^ MLV (Boehringer Ingelheim), ReproCyc^®^ PRRS-PLE (Boehringer Ingelheim), Ingelvac PRRSATP^®^ (Boehringer Ingelheim) and Fostera^®^ PRRS (Zoetis) were developed against PRRSV-2 and were mainly licensed in the United States and China. Currently, no evaluation has yet been conducted for Ingelvac PRRSFLEX^®^ EU and ReproCyc^®^ PRRS EU after their launch in 2015. However, existing evidence suggests that all previously licensed PRRS MLV vaccines of both genotypes 1 and 2 elicit only relatively weak humoral and cell-mediated immune (CMI) responses, as observed during infection with virulent PRRSV strains (Diaz et al., 2006; Zuckermann et al., 2007). Based on challenge experiments to evaluate vaccine efficacy, it appears that PRRSV-MLVs do confer late but effective protection against genetically homologous wild type PRRSV strains, while conferring only partial protection or no protection against heterologous strains (Charerntantanakul, 2012; Roca et al., 2012). These experimental observations are also consistent with reported atypical PRRS outbreaks in vaccinated herds since 1996 (Mengeling et al., 1998; Opriessnig et al., 2002), which indicate all MLVs currently used are ineffective and cannot meet practical needs.

Besides efficacy, safety is another concern for PRRSV-MLVs, as shedding and persistent MLV infections have been reported in vaccinated hosts. In fact, it has been demonstrated that MLV-vaccinated pigs can develop viremia for up to 4 weeks after immunization, leading to the spread of vaccine virus to naive animals (Charerntantanakul, 2012; Wang et al., 2013c). Consequently, reversion to virulence of PRRSV-MLVs is of great concern. Since Ingelvac PRRS^®^ MLV, the first licensed PRRSV vaccine, was widely used for vaccination on swine farms in both China and United States, field isolates from a later PRRSV outbreaks exhibited nearly identical nucleotide sequences to the vaccine strain were reported in both countries (Botner et al., 1997; Wang et al., 2010). Furthermore, evidence of recombination between MLVs and wild-type strains has been reported as well (Botner et al., 1997; Madsen et al., 1998; Wang et al., 2010; Wenhui et al., 2012). Therefore, practitioners of the swine industry are concerned about both efficacy and safety of current attenuated vaccines.

Another issue that is less known regarding current MLVs is the role of antibody-dependent enhancement (ADE) of infection. Soon after characterization of PRRSV, several studies demonstrated that non-neutralizing antibody (non-NA) was responsible for ADE during PRRSV infection in PAMs (Yoon et al., 1996, 1997). This phenomenon can be described as enhanced internalization of virus by macrophages as a result of opsonization mediated by non-NA. Studies focusing on epitopes responsible for inducing antibodies involved in ADE mapped them to both the N protein and GP5 of PRRSV (Yoon et al., 1996; Cancel-Tirado et al., 2004; Welch et al., 2004). Based on these findings, there are two potential issues about ADE that have not been addressed when using MLVs for vaccination. First, MLV evokes a delayed NA response, as observed for pathogenic PRRSV strains. Theoretically, it means that most PRRSV-specific antibodies produced within the first 4 weeks after MLV vaccination are non-NA and there might be a “window period” for potential ADE. However, little is known about whether these none-NAs at the earlier stage of immunization (before the appearance of NA) could actually induce ADE and exacerbate disease if either homogenous or heterogeneous PRRSV infection occurs during this “window period.” Secondly, since ADE of PRRSV could be mediated by sub-neutralizing antibody as well (Yoon et al., 1996, 1997), NA raised by one strain of MLV in immunized herds could serve as sub-neutralizing antibody (sub-NA) for a circulating or re-emerging heterogeneous virus. In this case, even 4 weeks after immunization (coinciding with the time of appearance of NA in vaccinated animals), NA specific for vaccine strains may still have an opportunity to induce ADE if the vaccinated herds were infected by a genetically and antigenically heterogeneous virus. In such a scenario, it is possible that MLV vaccination actually places vaccinated herds at increased risk for exacerbation of PRRS by sub-NA-mediated ADE. This type of ADE may be related to the presence of auto-anti-idiotypic antibodies that bind directly to NA antibodies against either PRRSV GP5 or M antigens; consequently, such antibodies may exacerbate PRRSV infection (Jiang et al., 2003; Zhou et al., 2004).

### Inactivated PRRSV Vaccines

In contrast to MLV vaccines, inactivated PRRSV vaccines have been licensed worldwide due to better safety. However, since 2005 these vaccines are no longer available in the United States due to their poor observed efficacy (Charerntantanakul, 2012). Meanwhile, several reports have partially explained the poor performance of inactivated PRRSV vaccines against wild type virus infection, with observations of a lack of detectable production of PRRSV-specific antibodies (neither non-NA nor NA) (Kim et al., 2011) and lack of CMI responses (Bassaganya-Riera et al., 2004; Piras et al., 2005). For PRRSV-specific immune responses induced by inactivated PRRSV vaccines for both genotypes (KV/ADJ, Progressis^®^, Merial Labs, PRRSV-1 and PRRomiSe^®^, Intervet, PRRSV-2), viral NA titers were generally below 8 and could not effectively clear virus (Nilubol et al., 2004; Zuckermann et al., 2007). Therefore, inactivated PRRSV vaccine failed to show statistically significant benefits in vaccinated herds against wild type PRRSV during challenge experiments (Scortti et al., 2007; Zuckermann et al., 2007). However, more recently a strategy using intranasally delivered nanoparticle-entrapped inactivated PRRSV vaccine along with poly(lactic-co-glycolic) acid or whole cell lysate of *Mycobacterium tuberculosis* as adjuvant elicited broadly cross-protective anti-PRRSV immunity against heterogeneous PRRSV strains (Binjawadagi et al., 2014a,b). Therefore, special formulations (nanoparticles) combined with novel adjuvants may enhance the immune response to inactivated PRRSV vaccines. However, it should be noted that conventional administration of inactivated PRRSV vaccine did confer some benefits in animals previously infected by wild type virus. These benefits included increased antibody production and CMI responses to infecting virus (Bassaganya-Riera et al., 2004; Kim et al., 2011) that were consistent with a report demonstrating that repeated exposure or long term administration of killed virus to seropositive sows boosted anti-PRRSV immunity and induced significant improvement in sow reproductive performance (Papatsiros et al., 2006). In summary, these data imply that inactivated PRRSV vaccine might exert a potential role as a therapeutic vaccine for PRRSV treatment rather than for disease prevention until novel formulations with adjuvants can be further developed.

### DNA, Subunit and Virus-Vectored PRRSV Vaccines

In addition to already licensed MLVs and inactivated vaccines, new approaches for administration of MLVs, such as co-administration with various adjuvants, use of DNA vaccines, subunit vaccines or virus-vectored vaccines incorporating other viruses, have been attempted. However, most vaccines developed using these techniques appear to be less effective than MLVs (Charerntantanakul, 2012). For example, soon after the first identification of PRRSV, baculovirus-expressed PRRSV structural proteins were tested as potential subunit vaccines. In the earliest investigation, swine immunized with a combination of insect cells expressing various PRRSV structural proteins only received partial protection (Plana Duran et al., 1997). Since then, a transgenic plant-based oral subunit vaccine against PRRSV was also tested (Chia et al., 2011). However, plant-based experimental vaccines have suffered from the same drawbacks as baculovirus-based subunit vaccines: limited efficacy in pigs (Renukaradhya et al., 2015a). Meanwhile, replication-deficient viruses (adenoviral vectors and poxvirus vectors) as vector vaccines have been tested for use with PRRSV with some success (Gagnon et al., 2003; Shen et al., 2007; Zheng et al., 2007; Jiang et al., 2008). However, even though mice immunized with adenovirus-based PRRSV vaccine exhibited high viral NA titers and strong lymphocyte proliferation responses (Gagnon et al., 2003; Jiang et al., 2008), similar experiments using recombinant adenovirus (rAd) in pigs have not yet been reported. For poxvirus vector-based PRRSV vaccines, vaccinated pigs challenged with virulent PRRSV had significantly lower body temperatures, lower levels of viremia and viral RNA load, but did not receive complete protection (Shen et al., 2007; Zheng et al., 2007).

Meanwhile, DNA vaccines have been tested against PRRSV as well, but still suffer the same drawbacks as the subunit or non-replicating virus-vectored vaccines. Moreover, little is known about whether DNA vaccines could adequately address the heterogenetic nature of PRRSV (Renukaradhya et al., 2015a). Although these new approaches for PRRSV vaccines are still require much development, they show promise as alternative methods for boosting MLV-induced protection. A recent study showed that pre-immunization with a DNA vaccine encoding truncated PRRSV N protein 2 weeks prior to MLV immunization led to improved PRRSV-specific immunity (increased NA titers and increased PRRSV-specific IFN-γ production), with reduced IL-10 and PRRSV-specific Treg production during the challenge experiment (Sirisereewan et al., 2017). Another study shows than immunization of pigs with a GP5 Mosaic T-cell DNA vaccine (codon-optimized mosaic sequences synthesized based on 748 independent PRRSV GP5 sequences) could evoke a higher virus-specific antibodies and IFN-γ mRNA expression, but still cannot confer full protection (Cui et al., 2016). Therefore, DNA vaccine based on a single PRRSV antigen may not enough for a complete protection. In summary, efficacy and safety concerns still surround current PRRSV MLVs. Consequently, vaccines based on new approaches are still far from ready for practical application and have little potential to replace MLVs without a major technological breakthrough. A list of licensed vaccine or vaccine candidates under development and their disadvantage or benefit was listed as **Table 2**.

**Table 2:** List of PRRSV vaccine or vaccine under development.

Vaccine type	Examples	Disadvantages/benefits
Modified live virus (MLV)	ReproCyc^®^ PRRS EU (*PRRSV-1*)	Partial or no protection to heterogonous strains; shedding of MLV and persistent infections; reversion to virulence; recombination between MLVs and wild-type strains/ complete protection for homogenous strain
	Ingelvac PRRS^®^ MLV (*PRRSV-2*)	
Inactive virus (KIV)	KV/ADJ, Progressis^®^(*PRRSV-1*)	Lack of detectable PRRSV-specific antibodies; lack of CMI responses; low NA titers (<8)/ long term administration confer benefit as therapeutic purpose
	PRRomiSe^®^(*PRRSV-2*)	
Subunit vaccine	Baculovirus expressed PRRSV proteins; transgenic plant-based oral subunit vaccine	Only partial protection
DNA vaccine	Plasmids DNA expressing PRRSV proteins	Same drawbacks as the subunit or non-replicating virus-vectored vaccines/ may be used to boost MLV-induced protection
Virus vectored vaccine	Poxvirus vector; Adenovirus vector	See benefits in swine challenge model, but not complete protection; adenovirus vector based vaccine has not been tested in swine yet

## The Heterogeneous Nature of PRRSV NAs

Although antibodies against PRRSV were initially considered as an ineffective component of the PRRSV-protective immune response or even deleterious due to the ADE concerns (Yoon, 1995), NAs have been considered effective against PRRSV (Lopez and Osorio, 2004; Charerntantanakul, 2012). This is consistent with the observation that inactivated PRRSV vaccines that failed to induce NA were not protective, while MLV which induced NA did confer protection to vaccinated animals against homologous PRRSV challenge (Lopez and Osorio, 2004; Charerntantanakul, 2012). Moreover, PRRSV-specific antibody kinetics suggested that the onset of NA after experimental infection correlates with clearance of the virus from the circulation and tissues (Labarque et al., 2000; Robinson et al., 2015).

In addition to the fact that genetic and antigenic variability among PRRSV isolates has hampered development of effective prevention or control strategies based on antibody-mediated virus neutralization (Kim et al., 2013), the major neutralizing targets among PRRSV antigens are still controversial. However, it is known that immune responses to PRRSV isolates are strain-specific (Mengeling et al., 2003). Soon after characterization of the PRRSV genome and identification of encoded ORFs, it was postulated that GP5, the major glycosylated envelope protein encoded by PRRSV-ORF5, acts as a major inducer of NA, as had been earlier observed for related viruses lactate dehydrogenase-elevating virus (LDV) and EAV (Zhang et al., 1998). Indeed, early reports investigating PRRSV-GP5-specific monoclonal antibodies (mAbs) identified specific epitopes of GP5 that correlated with viral neutralization (Pirzadeh and Dea, 1997; Zhang et al., 1998; Weiland et al., 1999). Based on mAb screening, a liner neutralizing epitope (designated the B epitope) and a non-neutralizing epitope (designated the A epitope) of VR-2332 GP5 protein were identified (Ostrowski et al., 2002; Plagemann et al., 2002). The core sequence of the B epitope was mapped to aa37-45 of GP5 and the antibody recognizing the B epitope was consistent with neutralizing activity of sera from VR-2332-infected pigs (Plagemann et al., 2002). Meanwhile, the hypothesis that GP5 is the major neutralization target of PRRSV was further supported by identification of neutralization-resistant mutants containing amino acid substitutions in GP5 or chimeric virus containing regions of ORF5 that had been swapped among virus strains that were susceptible or resistant to NAs (Kim and Yoon, 2008; Fan et al., 2015). Moreover, recent studies also indicated that GP5 regions that are highly variable among PRRSV strains, such as aa32-34, aa38-39 and aa57-59 regions within the N-terminal ectodomain of GP5, significantly influenced the susceptibility of the mutant viruses to NA (Kim et al., 2013). Thus, numerous lines of evidence support G5 as a major target of NAs.

In another line of research, M protein encoded by PRRSV-ORF6, an unglycosylated membrane protein of 18–19 kDa, had been shown to be important in virus assembly and budding (Conzelmann et al., 1993). M protein associates with GP5 through formation of heterodimers via a disulfide bond between the N-terminal ectodomains of both GP5 and M (Mardassi et al., 1995, 1996; Wieringa et al., 2004). Because a mAb against M protein is able to neutralize PRRSV infection (Yang et al., 2000), M protein has also been studied as a vaccine candidate. Indeed, immunization of pigs with both PRRSV-GP5 and M protein expressed by *M. bovis* BCG strain confers a certain degree of protection that correlates with appearance of NAs (Bastos et al., 2004). Notably, a recent study demonstrated that variation of a single amino acid (Tyr-10) in M protein confers virus resistance to pig serum with broad NA activity (Trible et al., 2015). Together, all of these data imply that the M protein of PRRSV also plays a role in viral neutralization in addition to the role played by GP5.

Conversely, several mAbs reacting with antigenic regions corresponding to the putative ”major neutralizing epitope” for PRRSV have been demonstrated to possess little neutralizing activity (Van Breedam et al., 2011). Moreover, one report demonstrated that PRRSV M-GP5 ectodomain-specific antibodies from PRRSV-neutralizing serum bound to virus but did not neutralize it (Li and Murtaugh, 2012). Therefore, it appears that antibody binding to ectodomain alone is not sufficient to ensure complete neutralization of PRRSV.

For European prototype strain Lelystad, a pepscan did not identify any virus-neutralizing epitopes in E, GP5 or M, while GP2, GP3 and GP4 were shown to possess neutralizing epitopes. In fact, GP3 appears to be the major target of NA from sera of Lelystad-infected pigs (Vanhee et al., 2011). It is important to point out that GP2, GP3 and GP4 are able to form a multi-protein complex that plays an important role in viral infectivity and receptor binding (Lee et al., 2004; Wissink et al., 2005; Das et al., 2011). Furthermore, GP4 of both the North American prototype strain VR-2332 and European prototype strain Lelystad contains a viral-neutralizing epitope and might be a driving force in PRRSV evolution (Weiland et al., 1999; Costers et al., 2010; Vanhee et al., 2010). A list of the function of all PRRSV proteins and their role in viral infection and neutralization was summarized in **Table 3**.

**Table 3:** List of PRRSV-ORFs, corresponding viral proteins and potential for virus neutralization.

ORFs	Proteins	Function	Mediating virus neutralization
ORF1a	nsp1α, nsp1β	Papain like cysteine protease (PLP), zinc-finger protein, antagonists for IFN induction and signaling (JAK/STAT pathway)	NA
	nsp2	PLP, deubiquitinase, IFN induction antagonist, transmembrane (TM) protein for replication complex	
	nsp3	TM protein for replication complex	
	nsp4	IFN induction antagonist	
	nsp5	TM protein for replication complex, antagonist for JAK/STAT signaling	
	nsp6	Predicted nsp, function unknown	
	nsp7α, nsp7β	Function unknown	
	nsp8	Function unknown, contains N-terminal domain of nsp9	
ORF1a’-TF	nsp2TF, nsp2N	PLPs	NA
ORF1b	nsp9	RNA-dependent RNA polymerase	NA
	nsp10	RNA NTPase/helicase; contains putative zinc-binding domain	
	nsp11	Uridylate-specific endoribonuclease, IFN induction antagonist	
	nsp12	Unknown	
ORF2a	GP2a	Minor glycosylated protein; essential for virus infection; forming complex with GP3-4; responsible for receptor binding	Yes, but only reported for PRRSV-1 Lelystad strain
ORF2b	E	Minor unglycosylated but myristoylated structural protein, essential for virus infection; forming complex; possesses ion-channel-like properties and may function as a viroporin in the envelope	NA
ORF3	GP3	Minor glycosylated structural protein; forming complex with GP2a and GP4 which is responsible for receptor recognition;	Yes
ORF4	GP4	Minor glycosylated structural protein; forming complex with GP2a-3-4 and responsible for receptor recognition	Yes, might be a driving force in PRRSV evolution
ORF5	GP5	Major glycosylated structural protein with a variable number of potential N-glycosylation sites	Yes, initially considered as major neutralizing target among all PRRSV structure proteins
ORF5a	GP5a	Minor unglycosylated protein; essential for virus viability;	No
ORF6	M	Forming heterodimer with GP5 which is crucial for virus infectivity; plays a key role in virus assembly and budding	Not sure, but mutation of Tyr10 of M results neutralization resistance mutant
ORF7	N	Component of the viral capsid; IFN antagonist	No

To date, the mechanism of antibody-mediated PRRSV neutralization is still unclear, due to conflicting data from various research studies (see **Table 1**). One plausible explanation for the discrepancies lies in the variability among PRRSV isolates used for antibody production; among the numerous studies, antigenic determinants and biological properties may differ radically (Trible et al., 2015). Supporting evidence for the heterologous nature of PRRSV neutralization stems from observations that the B epitope of GP5, a major linear neutralizing epitope for VR-2332 (Ostrowski et al., 2002; Plagemann et al., 2002), is no longer sufficient for neutralization of HP-PRRSV-HuN4 after mutation of a single amino acid of G5 (aa39) that is not predicted to affect antibody recognition (Leng C.L. et al., 2012). Moreover, in a study to systematically investigate neutralization susceptibility among PRRSV isolates, the presence of previously identified neutralizing epitopes among GP3, GP4 and GP5 did not correlate with the neutralization phenotype of the corresponding PRRSV isolates (Martinez-Lobo et al., 2011). A recent study focusing on the cross-reactivity of immune responses to PRRSV suggested that CMI and total antibody responses against PRRSV are broadly cross-reactive among PRRSV-2 isolates (Correas et al., 2017). However, NA titers are specific for the challenge isolate and homologous T cell responses show a positive association with homologous NA titers (Correas et al., 2017).

## Broad Neutralizing Epitopes in PRRSV: Inspiration From Discovery of HIV-1 Broad NAs

Our understanding of PRRSV envelope antigens and epitopes related to viral neutralization are inconclusive. Previous reports have demonstrated that cellular receptors for PRRSV interact with various PRRSV envelope proteins (Lunney et al., 2016). However, no crystal structure information is available that describes most PRRSV envelope proteins, which makes it difficult to understand the structural basis of the virus-receptor interaction or antibody-mediated neutralization. Furthermore, most structural data or models used for visualizing PRRSV envelope proteins (such as GP5) still rely on bioinformatic analysis or comparison with related virus counterparts (e.g., EAV) to predict putative domains or structures. Consequently, the predicted ectodomain or transmembrane regions that are used to evaluate neutralization sites among various studies are not in agreement (Thaa et al., 2013; Do et al., 2016). Moreover, most studies have used artificially synthesized peptides or bacterially expressed viral proteins to mimic authentic PRRSV envelope proteins for identification of PRRSV-neutralizing epitopes. However, these tools apparently do not mimic authentic proteins in terms of membrane association or proper protein folding in PRRSV virions. Of special note, conserved conformational epitopes or epitopes requiring post-translational modification, such as glycosylation, may exist among heterogeneous PRRSV isolates and are not reliably reconstructed using many conventional research models.

The highly heterogeneous nature of PRRSV and its CD163 dependency for host cell tropism is analogous to human immunodeficiency virus-1 (HIV-1) and its receptor CD4. Therefore, it is a reasonable to expect that antibodies recognizing certain conserved epitopes may play an essential role in broad PRRSV neutralizing effects. In HIV-1 research, HIV-1-infected donor serum samples exhibiting the ability to neutralize diverse primary strains of HIV-1 were documented as early as in 1993, about 10 years after discovery of HIV (Mascola and Haynes, 2013). Since then, only a handful of neutralizing human monoclonal antibodies (hmAbs) has been reported (Burton et al., 1994; Trkola et al., 1995). Analysis of epitopes recognized by these hmAbs revealed several unique regions of HIV-1 gp120 (the major envelope protein of HIV-1) that correlate with broad neutralization, such as the CD4-binding site of gp120 (hmAb b12) and surface glycans on the outer domain of gp120 (hmAb 2G12) (Mascola and Haynes, 2013). To date, analysis of available HIV-1 broad NAs suggests that the majority of them recognize peptidoglycan moieties located in the V1, V2 and V3 regions of gp120 or glycans located in the outer domain, and mimic the viral CD4 receptor-binding region via their complementarity-determining regions rather than a linear epitope (Mascola and Haynes, 2013; Eroshkin et al., 2014). In PRRSV, little is known with regard to how glycosylation sites located in GP5 or other envelope proteins, such as GP2, GP3 and GP4, are associated with antibody recognition sites. This lack of information is partly due to the lack of structural information regarding receptor interaction regions. Furthermore, no report yet exists that describes any PRRSV antibody from swine or mice that recognizes peptidoglycan on the virion envelope. In spite of this lack of information, most studies still suggest that glycosylation sites of PRRSV envelope proteins (especially GP5) play a role in PRRSV escape, blocking, or minimization of virus-neutralizing antibody responses rather than directly functioning as potential NA targets (Jiang et al., 2007).

Meanwhile, it is highly interesting that hosts infected with HIV develop antibodies against major envelope protein surface glycans (Calarese et al., 2003). Notably, crystal structures of the antibody-antigen complex revealed that the hmAb 2G12 binds a cluster of surface glycans and was shown to adopt an unusual domain swap configuration in which the two heavy chains interact to form a large monovalent binding surface (Calarese et al., 2003). Indeed, no antibody with a similar configuration had been described before that report. Furthermore, it was unclear how such an antibody was induced in the HIV-infected individual. Since no applicable technique currently exists to generate mAb in pigs (as was done for human mAbs), it is still unknown if antibodies recognizing glycan or peptidoglycan exist in PRRSV-infected pigs. However, it is reasonable to speculate that infected pigs produce PRRSV-specific broad NAs that recognize conserved epitopes (Doria-Rose et al., 2009; Simek et al., 2009). In fact, pig sera from swine herds exposed to circulating field PRRSV strains contained PRRSV-specific broad NAs against both PRRSV genotypes (Robinson et al., 2015).

In recent years, a combination of FACS and single cell isolation techniques makes it possible to isolate a single Ig-secreting B cell from peripheral blood mononuclear cells (PBMC) and obtain the cDNA sequence encoding paired light chain and heavy chain via single cell DNA sequencing (Tiller et al., 2008; Scheid et al., 2009a,b; Pan, 2014). These techniques has been successfully used for HIV-1 broad NA screening and production of recombinant antibodies in mammalian cells (Tiller et al., 2008; Scheid et al., 2009a,b; Pan, 2014). Considering the fact that PBMCs from PRRSV-infected swine are easy to isolate, similar techniques should be applied to PRRSV research for screening of Ig-secreting B cells with broad neutralizing activity from PRRSV-infected swine. Ultimately, if conserved epitopes for broad NAs can be characterized, it will impact the development of vaccines conferring broad protection against heterogeneous PRRSV strains.

## Reverse Genetic Based Chimeric PRRSV Strains to Broaden Cross-Protection

There is no agreement regarding a predominant PRRSV neutralizing target, as current data suggests PRRSV neutralization appears to be strain-specific. Therefore, other approaches have been used to broaden cross-protection of single strain-based MLVs. As the PRRSV genome is single-stranded and consists of positive-sense RNA, reverse genetics techniques have been used for construction of infectious cDNA clones. One strategy has been to generate chimeric virus by swapping gene segments encoding structural proteins from heterologous PRRSV strains. In one study, chimeric viruses containing shuffled GP3 genes from six different PRRSV strains were generated (Zhou et al., 2012). However, only one chimeric virus was able to induce significantly higher levels of cross-NAs in pigs against heterologous PRRSV strain FL-12, suggesting that shuffling of areas of a single structural protein may not be enough to induce cross-NAs against heterologous PRRSV. To support this explanation, in a modified study conducted by the same group, shuffling of both the GP4 and M genes from different parental viruses to create chimeric viruses did indeed broaden induction of heterologous cross-NAs (Zhou et al., 2013). Thus, this approach appears to be more promising than shuffling of a single ORF for inducing broad protection. Moreover, a recent study demonstrated that by shuffling even a greater number of structural genes (ORFs 3-6) from six heterologous PRRSV strains into a PRRSV-VR2385-based backbone, rescued chimeric viruses exhibiting improved cross-protective efficacy against multiple heterologous strains (Tian et al., 2017).

In addition to gene shuffling to generate chimeric virus strains, phylogenetic analysis offers another tool to understand genetic diversity of PRRSV and seek a common antigen-coding sequence among heterologous PRRSV strains. Using this approach, broadly protective candidate strains were generated to counter the extraordinary genetic diversity of PRRSV. After performing phylogenetic analysis with alignment of 59 non-redundant, full-genome sequences of type 2 PRRSV “centralized” sequences were identified that were of equal genetic distance to all 59 wild-type PRRSV strains (Vu et al., 2015). In this way, centralized sequences of these PRRSV isolates were computationally designed and synthesized to generate a novel infectious clone designated PRRSV-Con. Rescued PRRSV-Con virus from the infectious clone was viable and replicated effectively in MARC-145 cells; moreover, pigs infected with this virus exhibited expanded levels of heterologous protection (Vu et al., 2015). However, based on challenge data, PRRSV-Con was only able to confer slightly improved protection against the virulent MN184 strain. This protection was not enough to prevent disease and PRRSV-Con was itself virulent in host animals (Vu et al., 2015). Therefore, sequence optimization and attenuation of PRRSV-Con is further required to gain maximum heterologous protection and reduce host virulence [for detail of PRRSV phylogenetic analysis, please see following reviews (Murtaugh et al., 2010; Shi et al., 2010; Stadejek et al., 2013)]. In addition, a single strain containing “centralized” sequences may confer better protection, but cannot provide complete protection to numerous heterologous PRRSV strains. In summary, although PRRSV-Con is still far from being an effective vaccine candidate, the artificial design approach to generate novel PRRSV strains based on centralized sequences holds great promise as a new for future vaccine development.

## Interferon-Inducing PRRSV Strains and their Potential as DIVA Vaccine Backbone

### Interferon Induction and Signaling

In addition to antigenic and genomic variations among different PRRSV isolates, virus infection elicits typical immunological dysfunctions in PRRSV-infected pigs, including inhibition of innate immunity, delayed and low level production of NAs against PRRSV, as well as weak CMI responses (Albina et al., 1998; Labarque et al., 2000; Xiao et al., 2004). Interferons (IFNs), the major players that provide innate immunity against viral infection, are divided into three different types, I-III. Type I IFNs comprise the largest IFN family, which includes IFN-α, IFN-β, IFN-𝜀, IFN-κ and IFN-ω (Pestka et al., 2004; Uze et al., 2007). Although almost all cell types are able to produce IFN-α/β, plasmacytoid dendritic cells (pDC) are considered to be the major source for IFN-α during virus infection (Siegal et al., 1999; Liu, 2005). Type II IFN includes only IFN-γ, which is produced exclusively by activated T cells, natural killer cells and macrophages (Valente et al., 1992). IFN-γ plays a major role in establishing cellular immunity, but it is also capable of inducing expression of genes that respond to type I IFNs as well (Decker et al., 1989; Lew et al., 1989).

The induction of IFNs typically results from the activation of host pattern-recognition receptors (PRRs), such as RIG-I-like receptors (RLR), and Toll-like receptors (TLR) (Gonzalez-Navajas et al., 2012; Nan et al., 2014). Like other cytokines, IFNs stimulate cells via activation of the JAK/STAT pathway (Schindler et al., 1992; Yang et al., 2017). The cascade of events during activation of this pathway results in expression of IFN-stimulated genes (ISGs) (Katze et al., 2002), which include antiviral effectors to restrict virus replication. Besides their ability to inhibit virus replication, IFNs also exhibit anti-proliferative activity, stimulate cytotoxic T cells and modulate immune responses (Pestka, 2007).

### Antagonizing of IFN Induction and Signaling by PRRSV

It has been confirmed that PRRSV encodes several IFNs antagonists within its genome that block both IFN induction and IFN-activated JAK/STAT signaling (Patel et al., 2010; Wang and Zhang, 2014; Yang et al., 2017). The nsp1 of PRRSV self-cleaves to generate two subunits: nsp1α and nsp1β (Chen et al., 2010a). Both of these dramatically inhibit IFN-β expression by affecting the IRF3 signaling pathway (Chen et al., 2010a). Moreover, nsp2, the largest non-structural protein of PRRSV, inhibits IFN induction by blocking IRF3 phosphorylation and nuclear translocation via its cysteine protease domain (Li et al., 2010; Sun et al., 2010). PRRSV-nsp4 is another IFN antagonist that interferes with the NF-κB signaling pathway through the cleavage of NEMO, leading to down-regulation of IFN-β production induced by poly (I:C) (Huang et al., 2014). The nsp11, another IFN antagonist, is able to suppress activation of IFN-β by performing endoribonuclease cleavage of IPS-1 mRNA (Shi et al., 2011). Moreover, IFN-antagonizing activity is not restricted to PRRSV nsps. Structural proteins, such as the N protein, were found to inhibit IFN-β mRNA induction by poly (I:C) in immortalized PAM cells by interfering with phosphorylation and nuclear translocation of IRF3 (Sagong and Lee, 2011). In addition to inhibiting IFN induction, PRRSV nsp1β also inhibits IFN-activated JAK/STAT signaling by inducing degradation of KPNA1, which is a critical transporter protein that mediates nuclear import of ISGF3 (Patel et al., 2010; Wang et al., 2013b). Moreover, nsp7, nsp12, GP3 and N of PRRSV also interfere with IFN-activated signaling by unknown mechanisms (Wang et al., 2013a; Yang et al., 2017).

Numerous reports have confirmed that PRRSV infection *in vitro* could result in inhibition of IFN induction (via both RLRs and TLRs pathways) in a variety of cell types. Meanwhile, *in vivo* studies have suggested that certain PRRSV isolates (e.g., HP-PRRSV HuN4-F112) could induce some IFN-α secretion in infected swine (Liu et al., 2010; Dwivedi et al., 2012; Guo et al., 2013). However, no report examined IFN-β levels in PRRSV-infected swine due to the lack of availability of an ELISA kit for porcine interferon detection. Regarding pDC as the major source of IFN-α *in vivo*, a recent study suggested that IFN-α secretion during PRRSV infection *in vivo* might be a result of TLR7 activation through direct contact of PRRSV-infected macrophages with pDC, which does not require live virus or virus replication (Obdulio et al., 2015). Moreover, it was reported that infection of pDC by PRRSV blocks induction of IFN-α production by TLR9 agonist CpG (Baumann et al., 2013). Since PRRSV blocks the IFN-activated JAK/STAT pathway, low levels of bioactive IFN-α may not be enough to activate the antiviral response (Patel et al., 2010; Yang et al., 2017). Furthermore, although type I IFNs induction typically results from activation of PRRs, IFNs are not the only cytokines produced if PRRs are activated (Nan et al., 2014); thus, IFNs alone might not be sufficient to activate host innate immunity. Ultimately, a synergistic effect mediated by both IFNs and other pro-inflammatory cytokines might be needed to fully activate the host immune response. This speculation is consistent with a report demonstrating that pretreating swine with IFN-α prior to challenge eased PRRS signs, but was unable to prevent pigs from dying; however, survival times were extended (Dong et al., 2012).

One strategy to enhance PRRSV-induced protective immunity is to activate the innate immune response using TLR agonists. It has been shown that activation of TLR7 by immunization with inactive PRRSV vaccine induced high levels of PRRSV-specific humoral immune responses and T lymphocyte proliferation in mice (Du et al., 2016). Similar results were observed when pigs were immunized by inactive vaccine along with TLR3 and TLR7/8 ligands (Zhang et al., 2013). Moreover, various cytokines and TLR ligands have been tested as adjuvants to enhance PRRSV vaccine-induced immunity (Charerntantanakul, 2009). However, as most cytokines and TLR ligands in these studies play a natural role in host innate immunity, they would be more effective in conjunction with new vaccine design if inhibition of innate immunity by PRRSV could be mitigated.

### IFN-Inducing PRRSV Isolates

Recently, two PRRSV strains that uniquely induce type I IFNs production have been tested for their potential as vaccine candidates (Nan et al., 2012; Ma et al., 2016; Sun et al., 2016). One of them, PRRSV-A2MC2, a moderately virulent strain, shares high nucleotide identity with prototype VR-2332; sequencing analysis has demonstrated that A2MC2 is closely related to VR-2332 and Ingelvac PRRS^®^ MLV, with an identity of 99.8% at the nucleotide level. PRRSV-A2MC2 was the first reported novel strain with strong ability to induce IFN synthesis in cultured cells (Nan et al., 2012; Wang et al., 2013c). Moreover, A2MC2 induces IFNs in both MARC-145 and PAM cells and viral replication is required for IFN induction in infected cells (Nan et al., 2012). While secretion of IFN-α2 and elevation of ISGs have been detected in A2MC2-infected MARC-145 cells, the mechanism of A2MC2 induction of IFNs is not well understood. The first 4.6 kb of the A2MC2 genome (including coding regions for nsp1α, nsp1β and nsp2) is identical to the VR-2332 sequence, but VR-2332 inhibits IFN induction. Recently, an infectious clone for A2MC2 was constructed by swapping genetic segments of A2MC2 with those of IFN-inhibitory PRRSV strain VR-2385. The results demonstrated that the middle half of the A2MC2 genome is responsible for IFN induction (Ma et al., 2017). Notably, A2MC2 infection of pigs resulted in earlier onset and higher levels of neutralizing antibody against homologous and heterologous strains than observed for MLV (Wang et al., 2013c).

Since A2MC2 is still moderately virulent, *in vitro* attenuation was conducted in MARC-145 cells by up to 90 serial passages (Ma et al., 2016). The resulting strain, A2MC2-P90, retains the ability to induce IFNs in cell culture and induces higher levels of NAs, but is as avirulent as MLV. Notably, during the passaging of A2MC2 in MARC-145 cells, a spontaneous deletion of 543 nucleotides (nt2994 to 3536) in ORF1a was observed relative to wild type A2MC2 virus that results in deletion of 181 amino acid residues from the hypervariable region of nsp2 (Ma et al., 2016). In a pig study using heterologous PRRSV challenge, A2MC2-P90 did not exhibit virus shedding, but was able to protect pigs against challenge with VR-2385 (92.3% nucleic acid identity to A2MC2) and also reduced nasal shedding of highly virulent MN184 (84.5% nucleic acid identity to A2MC2) (Fontanella et al., 2017). This result is encouraging, as a non-shedding MLV is an ideal vaccine for PRRSV control. Furthermore, this vaccine even prevented nasal shedding for a high challenge dose of MN184 (5 x 10^5^ TCID50). In summary, these data suggest that A2MC2-P90 might be used as a novel backbone for genetic element swapping or shuffling due to its unique features, such as ability to induce IFN, avirulence in swine and nsp2 deletion.

Another PRRSV strain with the ability to induce IFN synthesis is PRRSV-Con, the artificially synthesized infectious clone based on 59 wild-type PRRSV sequences (Vu et al., 2015; Sun et al., 2016). Although PRRSV-Con is still virulent for swine, it is able to induce type-I IFNs in cell culture, which is very similar to observations for A2MC2 (Sun et al., 2016). Interestingly, unlike A2MC2, the genetic determinant of the IFN-inducing phenotype for PRRSV-Con was mapped to the first 3.3 kb of the genomic fragment, which encodes nsp1α, nsp1β and the N-terminal part of nsp2 (Sun et al., 2016). All of these nsps are well-known IFN antagonists among PRRSV proteins (Sun et al., 2016). Although no further information is available, these data suggest that genetic determinants for IFN-induction in PRRSV-Con and A2MC2 differ. Theoretically, a chimeric virus strain containing both sequence segments responsible for IFN induction may therefore exhibit unprecedented ability to induce IFN synthesis both *in vitro* and *in vivo*.

Taken together, these data suggest that an attenuated PRRSV strain with the ability to elicit innate immunity during immunization may be a more favorable vaccine candidate than other strategies, such as inactive and DNA vaccines and immune stimulators (TLRs ligands or recombinant cytokines). Therefore, results gained from the study of A2MC2 and PRRSV-Con should guide future vaccine development against PRRS.

### PRRSV A2MC2-P90 as a Novel DIVA Vaccine Backbone

Currently, commercial PRRSV ELISA kits (such as IDEXX PRRS X3) are only capable of recognizing PRRSV-specific antibodies. Without isolation of viral RNA followed by sequencing identification, such tests cannot determine whether PRRSV-specific antibody conversion is caused by infection with wild type (WT) virus or by vaccination with MLV. Therefore, a DIVA (differentiation of infected and vaccinated animals) vaccine of PRRSV could be highly beneficial for PRRSV surveillance. During serial passaging of A2MC2 in MARC-145 cells, a spontaneous deletion of 543 nucleotides (nt2994-3536, aa934-1115 of pp1a) in ORF1a was observed relative to wild type A2MC2 virus, leading to a deletion of 181 amino acid residues within the hypervariable region of nsp2 (Ma et al., 2016). Screening of virus from different passages indicated that this deletion occurred in P60 and quickly became predominant by P62. Based on literature searches, a similar deletion in nsp2 (435 nucleotides, nt3080-3506, corresponding to aa961-1107 of pp1a) had been reported in PRRSV VR-2385 (Ni et al., 2011). Subsequently, an *in vivo* study of VR-2385 suggested that deletion of nsp2 does not have an effect on PRRSV virulence, but may be related to increased replication efficiency *in vitro* (Ni et al., 2011). Meanwhile, a MLV vaccine (TJM strain) developed against the Chinese strain HP-PRRSV was attenuated after 92 serial passages in cell culture. Upon examination of virus isolated from each passage, a spontaneous deletion of 120 amino acids (360 nucleotides, corresponding to aa628–747 for nsp2 of VR2332) within nsp2 occurred by passage 19 (Leng X. et al., 2012). Thus, it appears that the hypervariable region of PRRSV nsp2 is highly flexible for deletion, since deletions within this region were frequently reported for both field isolates (HP-PRRSV, PRRSV-1 Lena) and cell-adapted PRRSV strains (PRRSV2-TJM, VR-2385 and A2MC2-P90). However, it is interesting that deletions of hundreds of nucleotides in nsp2 were only observed in tissue culture-adapted PRRSV strains, but not in field isolates (Tian et al., 2007; Karniychuk et al., 2010). The deletion of 543 nucleotides in A2MC2-P90 is the largest spontaneous deletion ever reported in nsp2, which suggests greater deletion lengths could be artificially achieved using reverse genetics. Since several B-cell epitopes of PRRSV-nsp2 have been reported that occur within the deleted region mentioned above (de Lima et al., 2006; Chen et al., 2010b), the absence of B-cell epitopes corresponding to the deleted region may provide a novel negative serological marker that would aid the development of a DIVA assay (Renukaradhya et al., 2015b). Meeting this goal would satisfy one of the key criteria proposed in Colloquium on Prospects for Development of an Effective PRRS Virus Vaccine (2007) for improved PRRSV vaccine development. However, since this region is hypervariable among different PRRSV strains, one obstacle for utilizing this deletion for a negative serological marker is the development of a standard test to evaluate host B cell responses to epitopes in this region. Alignment of this region of various strains of the PRRSV-2 lineage shows it is quite variable. Therefore, insertion of artificial epitopes such as FLAG tags in this region might be an alternative to DIVA vaccine strategies to introduce a positive marker (as long as the insertion does not affect the biological function of nsp2). Further investigation is needed to address this as a potential strategy.

## Conclusion and Perspectives

Three decades have passed since the emergence of PRRS in 1987. Unfortunately, even with sustained efforts to understand PRRSV pathogenesis and vaccinology, an effective vaccine to prevent PRRSV has yet to be successfully developed. Concerns about safety of MLVs as vaccines have persisted in addition to concerns regarding the inability of MLVs to prevent new outbreaks. DNA vaccines, subunit vaccines or virus-vectored vaccines have all been tested, but their potential value as replacements for PRRSV-MLVs currently in use remains uncertain. Unfortunately, 10 years after the Colloquium on Prospects for Development of an Effective PRRS Virus Vaccine in 2007, a successful vaccine meeting all criteria set in that meeting is still not available. Therefore, single strain-based MLVs are still the only choice we have to control evolving PRRSV at the current time.

Fortunately, ongoing research has provided clues to guide creation of strategies to meet the 2007 goals mentioned above. A2MC2-P90, an attenuated strain developed via passaging in cell culture, shows promise because it shares a unique IFN-inducing phenotype with its parental strain. It also has a unique deletion in the nsp2 region that could be helpful for use as a negative DIVA vaccine marker. In addition, it is avirulent in swine and does not undergo virus shedding. Thus, A2MC2-P90 could serve as an ideal backbone for vaccine development. Alternatively, a novel hybrid PRRSV strain could be developed using DNA shuffling or artificial synthesis of PRRSV structural protein gene segments based on phylogenetic analysis (the method used for PRRSV-Con). As a further consideration, cross-protection capability of this novel hybrid against heterogeneous PRRSV could be included in the design if conserved epitopes of broad NA could be identified and characterized. Hopefully, a PRRSV vaccine designed using a combination of advanced techniques will be available that exhibits superior efficacy and safety to current vaccines.

## Author Contributions

All authors listed have made a substantial, direct and intellectual contribution to the work, and approved it for publication.

## Disclaimer

Mention of trade names or commercial products in this article is solely for the purpose of providing specific information and does not imply recommendation or endorsement.

## Conflict of Interest Statement

The authors declare that the research was conducted in the absence of any commercial or financial relationships that could be construed as a potential conflict of interest.
